# Clinical Characteristics and In-Hospital Course of Symptomatic Intracranial Hemorrhage After Intravenous Alteplase in Older Adults with Acute Ischemic Stroke: A Retrospective Cohort Study

**DOI:** 10.3390/neurolint18100194

**Published:** 2026-10-15

**Authors:** Sorina Nicoleta Munteanu, Dana Tutunaru, Aurel Nechita, Mihai Cristian Marinescu, Claudiu Elisei Tanase, Ioana Navalici, Dorel Firescu, Mihaela Moisei, Adrian Stan, Aurelia Romila

**Affiliations:** 1 Research Centre in the Medical-Pharmaceutical Field, Faculty of Medicine and Pharmacy, “Dunarea de Jos” University of Galati, 800008 Galati, Romania; sorina.munteanu@ugal.ro (S.N.M.); dana.tutunaru@ugal.ro (D.T.); aurel.nechita@ugal.ro (A.N.); cardiologmihaimarinescu@gmail.com (M.C.M.); tanaseclaudiumd@gmail.com (C.E.T.); navalio@gmail.com (I.N.); dorel.firescu@ugal.ro (D.F.); aurelia.romila@yahoo.com (A.R.); 2 “Saint Andrew the Apostle” Clinical Emergency County Hospital, 800578 Galati, Romania; 3 “Saint John” Clinical Emergency Hospital for Children, 800487 Galati, Romania

**Keywords:** acute ischemic stroke, intravenous alteplase, symptomatic intracranial hemorrhage, older adults, NIHSS, atrial fibrillation, Firth regression, in-hospital course

## Abstract

**Background/Objectives**: This study describes symptomatic intracranial hemorrhage (sICH) after intravenous alteplase in older adults with acute ischemic stroke. **Methods**: This retrospective single-center cohort included 95 unique patients aged ≥65 years treated in Galati, Romania, in 2020–2024. The binary hemorrhage variable was verified against clinical and computed tomography (CT) records. Study-defined sICH required new or increased CT hemorrhage with attributed deterioration, comprising a ≥4-point National Institutes of Health Stroke Scale (NIHSS) increase or other clinically relevant worsening. Comparisons and Firth models were exploratory, with admission NIHSS adjustment. **Results**: All 14 recorded hemorrhages met this definition (14.7%; 95% CI 9.0–23.2%): 10 met the NIHSS threshold and four had relevant worsening below it. Ten were detected before 24 h and four at the 24–48 h range; morphology included hemorrhagic infarction and parenchymal hematoma. All adjusted intervals included the null. Median hospitalization was five days longer with sICH. The mortality difference was 2.6 percentage points (conservative exact 95% CI range from −31.6 to 45.9). **Conclusions**: Adjusted association estimates are inconclusive and do not justify altering guideline-based eligibility. Prospective multicenter studies require standardized definitions, stroke-severity measures, reperfusion data, and functional outcomes.

## 1. Introduction

Intravenous thrombolysis remains an established reperfusion treatment for eligible patients with acute ischemic stroke. European Stroke Organisation and American Heart Association/American Stroke Association recommendations support intravenous alteplase when treatment criteria are fulfilled, and advanced age alone is not a reason to withhold therapy [1,2]. Pooled individual-patient data support alteplase use in appropriately selected patients older than 80 years [3], and registry studies have examined patients aged 90 years or older [4]. Stroke severity and cardiovascular comorbidity remain relevant to the safety assessment in older populations [3,5].

Symptomatic intracranial hemorrhage is an important early complication after reperfusion therapy. Reported frequencies vary with the clinical and radiological definition, study population, and imaging surveillance [6]. Blood–brain barrier injury and reperfusion-related inflammation provide biological context [7,8,9], but these mechanisms cannot be inferred from routine demographic variables.

Clinical studies and evidence syntheses have evaluated numerous characteristics associated with hemorrhage after thrombolysis. Age, admission neurological severity, atrial fibrillation, blood pressure, diabetes, glucose concentration, early ischemic imaging abnormalities, and other factors have been incorporated into observational analyses and risk models [10,11].

Admission NIHSS is particularly relevant because it provides a standardized measure of neurological severity and is repeatedly represented in post-thrombolysis hemorrhage prediction models [11].

The relationship between sex and hemorrhagic complications is less consistent. A systematic review and meta-analysis of thrombolysis studies found no overall sex difference in sICH, despite recognized sex-related differences in stroke phenotype, age, comorbidity, treatment characteristics, and functional outcome [12].

Contemporary cohort and trial analyses likewise indicate that observed sex differences may reflect accompanying clinical characteristics rather than a uniform sex-specific response to thrombolysis [13,14]. Atrial fibrillation also warrants attention in older adults because of its high prevalence and its association with cardioembolic stroke phenotypes and hemorrhagic outcomes in broader reperfusion populations [10,15].

The primary objective was to describe the frequency of sICH in adults aged 65 years or older treated with intravenous alteplase in this center. Detection timing and ECASS morphology further characterized the endpoint. Secondary objectives were to compare baseline characteristics and describe hospitalization and mortality according to sICH status.

Exploratory Firth models evaluated age, sex, and atrial fibrillation, with admission NIHSS added in Model B; these covariates were selected before the examination of this study’s results on clinical grounds. The primary analysis estimated a local frequency without a directional hypothesis. Secondary analyses examined exploratory group differences and associations rather than individual risk prediction.

## 2. Materials and Methods

### 2.1. Study Design and Setting

This retrospective, single-center observational cohort study analyzed index-hospitalization records of adults aged 65 years or older with acute ischemic stroke who received intravenous alteplase at the “Saint Andrew the Apostle” Clinical Emergency County Hospital, Galati, Romania, between 1 January 2020 and 31 December 2024.

This study used clinical and imaging information recorded during routine hospital care. No research-specific patient contact, therapeutic intervention, or modification of clinical management was introduced for the purposes of the present analysis.

The analytical unit was the individual patient and the index hospitalization. Reporting followed the Strengthening the Reporting of Observational Studies in Epidemiology recommendations for observational cohort studies. The completed STROBE Statement Checklist is provided in the Supplementary Materials.

### 2.2. Source Population and Cohort Derivation

The hospital admission registry and internal ischemic stroke diagnostic coding identified all 200 consecutive acute ischemic stroke hospitalizations between 1 January 2020 and 31 December 2024. This number represented the center’s recorded case volume during the five-year period; no additional source-population restriction was applied according to stroke severity, presentation time, or thrombolysis eligibility. Sixty hospitalizations involved patients younger than 65 years. Among the remaining 140 age-eligible hospitalizations, 42 did not involve intravenous alteplase, leaving 98 treated hospitalizations for patient-level deduplication.

Three records represented subsequent eligible hospitalizations of individuals who already had an earlier qualifying admission. Patient-level deduplication retained the earliest eligible hospitalization as the index record, resulting in 95 unique patients. Direct admissions and interhospital transfers were eligible when alteplase was administered at the study center. Patients who received thrombolysis at another institution before transfer were not included in the treated analytical cohort.

The age threshold of 65 years was prespecified within the approved geriatric-stroke research protocol before examination of this study’s results. It defined the project’s older-adult population and was not chosen post hoc to retain a particular sample size or hemorrhage frequency. It is a cohort eligibility criterion, not a thrombolysis treatment cutoff.

The entire eligible cohort was analyzed without sampling or a prospective sample-size calculation. sICH status did not influence inclusion. The same 95-patient source cohort was used in a separate publication on pre-thrombolysis biological correlates of admission Glasgow Coma Scale (GCS) [16]. Cohort derivation and some baseline variables overlapped, including age, sex, neurological severity, and clinical history. Here, tables and association models are organized around post-alteplase sICH and hospital course rather than GCS categories or biomarker associations. The earlier GCS correlation and proportional-odds analyses are not reproduced, and the shared cohort must not be interpreted as an independent replication.

### 2.3. Intravenous Thrombolysis and Post-Thrombolysis Monitoring

All 95 patients received intravenous alteplase at the study center following a decision by the treating stroke team under the local acute stroke protocol. The regimen was 0.9 mg/kg, capped at 90 mg, with 10% administered as an intravenous bolus and 90% infused over 60 min. Blood pressure was required to be below 185/110 mmHg before treatment and maintained below 180/105 mmHg during the first 24 h. The dose regimen and blood pressure targets were consistent with contemporary European and AHA/ASA recommendations [1,2].

Mechanical thrombectomy was not available as a verifiable patient-linked analytical variable and was therefore not included in the present analyses.

Antiplatelet and anticoagulant treatment was withheld during the first 24 h and initiated thereafter only following CT exclusion of hemorrhage. Routine non-contrast CT was scheduled at approximately 24 h, with immediate imaging after neurological deterioration. All 95 patients underwent at least one follow-up CT within 48 h. Minor deviations of several hours from the scheduled examination occurred.

Prior anticoagulant exposure was classified as vitamin K antagonist or direct oral anticoagulant (DOAC) therapy. Available details were aggregate summaries of agent class, last-dose timing, renal function, coagulation tests, and reversal. For vitamin K antagonist users, the recorded pre-alteplase INR values were ≤1.7. Calibrated anti-Xa and dabigatran-specific activity assays were unavailable at the center, and INR or activated partial thromboplastin time (aPTT) were not treated as specific measures of DOAC activity. Because these anticoagulant details were available only in aggregate form and were not linked to Study_ID, they were used descriptively and were not related to individual sICH outcomes.

### 2.4. Clinical Variables

Baseline characteristics included age, sex, residence, admission NIHSS, previous stroke, and recorded carotid or vertebrobasilar territory. Hypertension, diabetes, congestive heart failure, myocardial infarction, transient ischemic attack, and cancer history were coded YES/NO from documented clinical diagnoses, without imposing new retrospective laboratory or diagnostic thresholds. Atrial fibrillation (AF) included both a known pre-stroke diagnosis and AF detected during the index admission. Obesity indicated body mass index (BMI) ≥30 kg/m^2^ or a documented clinical diagnosis; smoking indicated current smoking. Alcohol use indicated documented daily or almost-daily consumption or explicit clinical notation of significant chronic use or abuse; no standardized questionnaire or quantitative weekly threshold was applied.

Prior antithrombotic exposure indicated chronic antiplatelet or anticoagulant treatment before the stroke. Separate binary flags identified each class; their union defined any exposure and their intersection identified both-class exposure. These flags describe recorded exposure rather than verified simultaneous ingestion. Territory indicators retained their recorded binary classifications and did not quantify infarct volume; one patient had neither territory flag recorded as positive.

The National Institutes of Health Stroke Scale (NIHSS) at admission was available for all 95 patients and represented baseline neurological severity. Age and admission NIHSS were analyzed continuously, scaled per 10-year and 5-point increase, respectively, in the Firth models. NIHSS measured at deterioration was used only for sICH classification and was not entered as a baseline covariate.

Hospital length of stay and in-hospital mortality were evaluated as downstream features of index hospitalization. They were used to characterize the clinical course according to sICH status and were not entered as antecedent predictors in the multivariable models.

### 2.5. sICH Definition and Radiological Classification

The primary outcome was study-defined sICH after intravenous alteplase. The original clinical variable, “Intracranial_Hemorrhage”, was coded YES/NO by the treating neurologist from the follow-up CT report. Its analytical field was rtPA_Complication_Intracranial_Hemorrhage. Coding was checked retrospectively against CT reports, followed by a review of the original clinical notes, CT images, and neurological course for all 14 positive cases. The prespecified sICH definition required new or increased hemorrhage on CT and acute neurological deterioration attributed to the hemorrhage by the treating team: either a ≥4-point NIHSS increase from the best recorded post-thrombolysis status or another clinically relevant worsening. Attribution was required for deterioration below the NIHSS threshold, rather than temporal coexistence alone.

CT hemorrhage without qualifying neurological deterioration did not meet the primary endpoint. European Cooperative Acute Stroke Study (ECASS) morphology was classified as HI1, HI2, PH1, or PH2 [6]. HI1 and HI2 represent petechial hemorrhagic infarction without hemorrhage-related mass effect; ischemic edema was considered separately. Symptomatic status and morphology were complementary classifications. The study definition was not presented as identical to ECASS II, NINDS, or SITS-MOST criteria.

Available post-thrombolysis imaging was assessed by two neurologists and one radiologist as part of the structured retrospective review. For the 14 hemorrhage-positive cases, the two neurologists independently reevaluated the CT images, with discrepancies resolved by consensus and radiological review available when required. Remote hemorrhage outside the ischemic territory was assessed separately. Detection timing was reported in the validated aggregate categories of <24 h, 24–48 h, and >48 h after alteplase administration; these categories describe the first positive CT detection window and should not be interpreted as the exact time of hemorrhage onset.

### 2.6. Data Handling and Analytical Framework

Direct patient identifiers were not retained in the analytical dataset. Repeated eligible hospitalizations were resolved at patient level before statistical analysis, with the earliest qualifying admission retained as the index hospitalization. Each patient contributed only one hospitalization.

Patient-level analyses were restricted to variables with verifiable linkage to the cohort. Variables reported in Table 1, hospital-course comparisons, and Models A/B were complete for all 95 patients; no imputation was used. Pre-stroke disability, baseline blood pressure, laboratory measures, and treatment intervals in a separate extract were excluded because their linkage to Study_ID could not be verified. Aggregate anticoagulant and hemorrhage-adjudication summaries were used descriptively, without assigning records to patients by row order. A legacy late-hemorrhage flag was excluded from endpoint classification.

The analytical framework separated antecedent demographic, cardiovascular, neurological, and pre-stroke treatment characteristics from sICH as the primary post-treatment endpoint. Length of stay and in-hospital mortality were analyzed separately as downstream hospital-course characteristics.

### 2.7. Statistical Analysis

Continuous and ordinal variables were summarized using the median and interquartile range; categorical variables were reported as frequencies and percentages. Between-group comparisons used the Mann–Whitney U test for continuous or ordinal variables and Fisher exact test for categorical variables.

Median differences (sICH minus no sICH, or women minus men for the within-sICH NIHSS comparison) were accompanied by percentile bootstrap 95% confidence intervals from 10,000 within-group resamples. Binary comparisons used exact conditional odds ratios with exact 95% confidence intervals. A Wilson interval described sICH frequency. For the mortality risk difference, a conservative exact 95% interval was constructed by combining two 97.5% Clopper–Pearson binomial intervals: the lower limit was the sICH lower bound minus the no-sICH upper bound, and the upper limit was the sICH upper bound minus the no-sICH lower bound. Bonferroni coverage was at least 95%.

Baseline unadjusted comparisons were considered exploratory. Because these comparisons were intended to characterize the clinical profile rather than test a confirmatory family of hypotheses, no multiplicity-adjusted significance threshold was applied.

Given 14 events, adjusted association estimation used Firth logistic regression with Jeffreys-prior penalization. Model A included age, female sex, and AF; Model B additionally included admission NIHSS. These covariates were selected before examination of this study’s results on clinical grounds: age and sex characterize demographic differences, AF represents a common cardiovascular condition associated with reperfusion outcomes, and admission NIHSS measures stroke severity [10,11,12,15]. No automated, stepwise, or outcome-driven selection was used. Adjusted odds ratios were reported with profile penalized likelihood 95% confidence intervals and likelihood-ratio *p* values against the null coefficient.

These models estimated exploratory associations rather than validated predictions or causal effects. An exploratory within-sICH comparison assessed admission NIHSS between women and men; NIHSS represented neurological severity and was not treated as measured infarct volume. Statistical calculations were reproduced using Python 3.12.14, NumPy 2.3.5, and SciPy 1.17.0; figures used Matplotlib 3.10.8.

### 2.8. Ethics

This study was conducted in accordance with the Declaration of Helsinki. The Ethics Committee of “Saint Andrew the Apostle” Clinical Emergency County Hospital, Galati, Romania, approved retrospective secondary analysis of routine-care records in 2020–2024 (approval no. 14225, 6 July 2026). Research-specific extraction began in July 2026 after approval. Original records remained in the hospital’s access-controlled medical-record system. Research exports were pseudonymized by removing direct identifiers and stored on a secured server with access restricted to the study team.

Individual informed consent was waived for this retrospective secondary analysis of pseudonymized routine-care records because this study involved no direct patient contact or research-specific intervention.

## 3. Results

### 3.1. Cohort Construction and sICH Frequency

**Figure 1:**
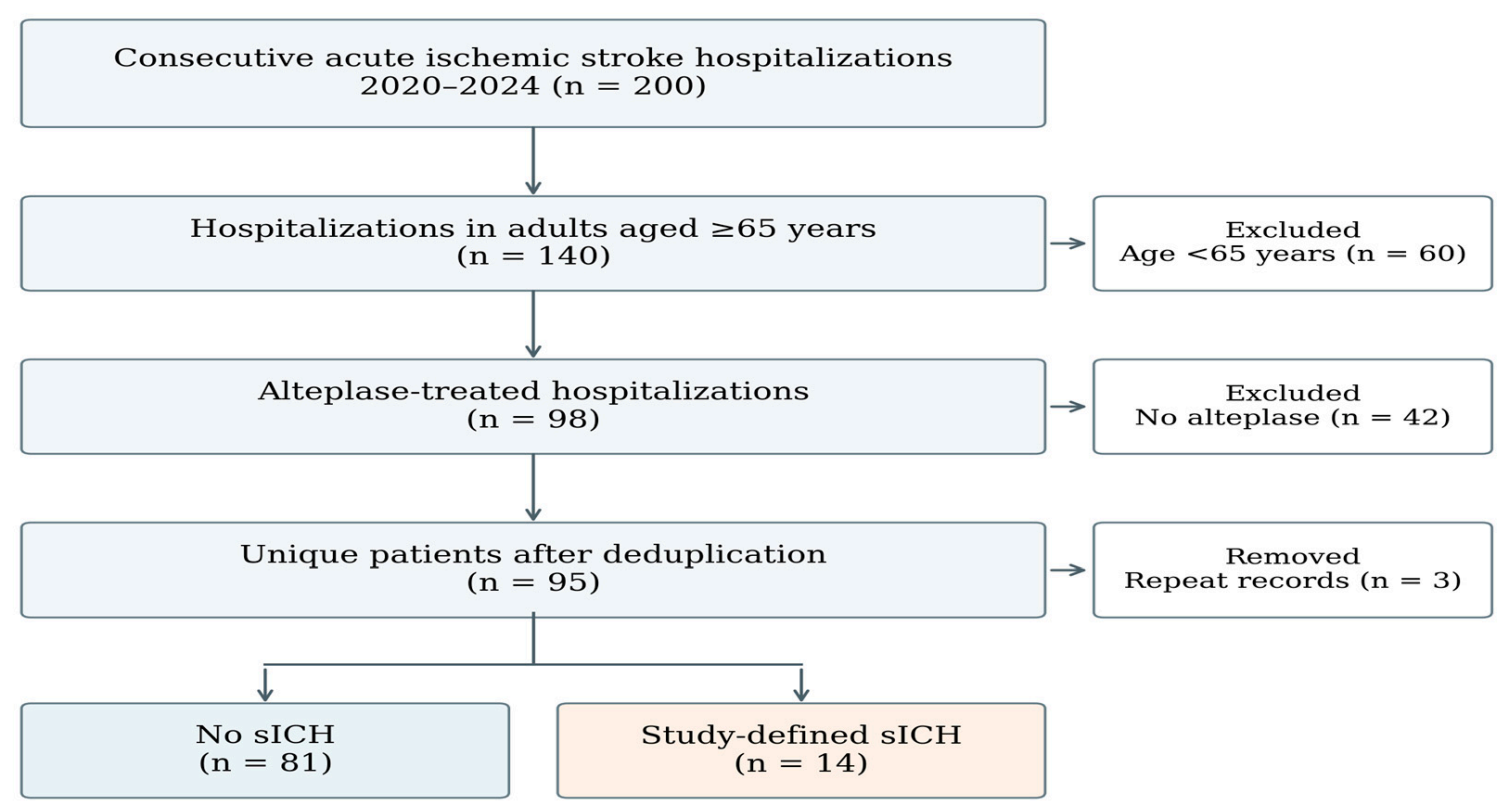
Study cohort flowchart from the source population to the final patient-level analytical cohort. Numbers refer to hospitalizations until patient-level deduplication and to unique patients thereafter. sICH, symptomatic intracranial hemorrhage.

The derivation of the analytical cohort from the consecutive acute ischemic stroke hospitalizations, including the age restriction, alteplase treatment, patient-level deduplication, and final sICH classification, is summarized in Figure 1.

From 200 consecutive acute ischemic stroke hospitalizations, 140 involved patients aged ≥65 years and 98 age-eligible hospitalizations involved intravenous alteplase. After removal of three repeat eligible admissions, the analytical cohort comprised 95 unique patients. Fourteen patients met the study-defined sICH endpoint, corresponding to 14.7% (Wilson 95% CI 9.0–23.2%), while 81 had no sICH. No asymptomatic intracranial hemorrhage was recorded.

Among 24 patients with prior anticoagulant exposure, the aggregate summary identified 17 DOAC users and seven vitamin K antagonist users. Exact last-dose timing was available for 18/24 anticoagulant-exposed patients and for 11/17 DOAC users.

Among DOAC users, eight had a documented interval >48 h and nine had a reported interval ≤48 h, comprising three exact and six estimated intervals. Drug-specific DOAC activity assays were not performed, and no anticoagulant reversal agent was administered before alteplase.

Because these details were available only in aggregate form, they could not be linked to individual sICH outcomes or used to independently verify treatment eligibility for specific patients.

### 3.2. Timing and Radiological Characteristics of sICH

**Figure 2:**
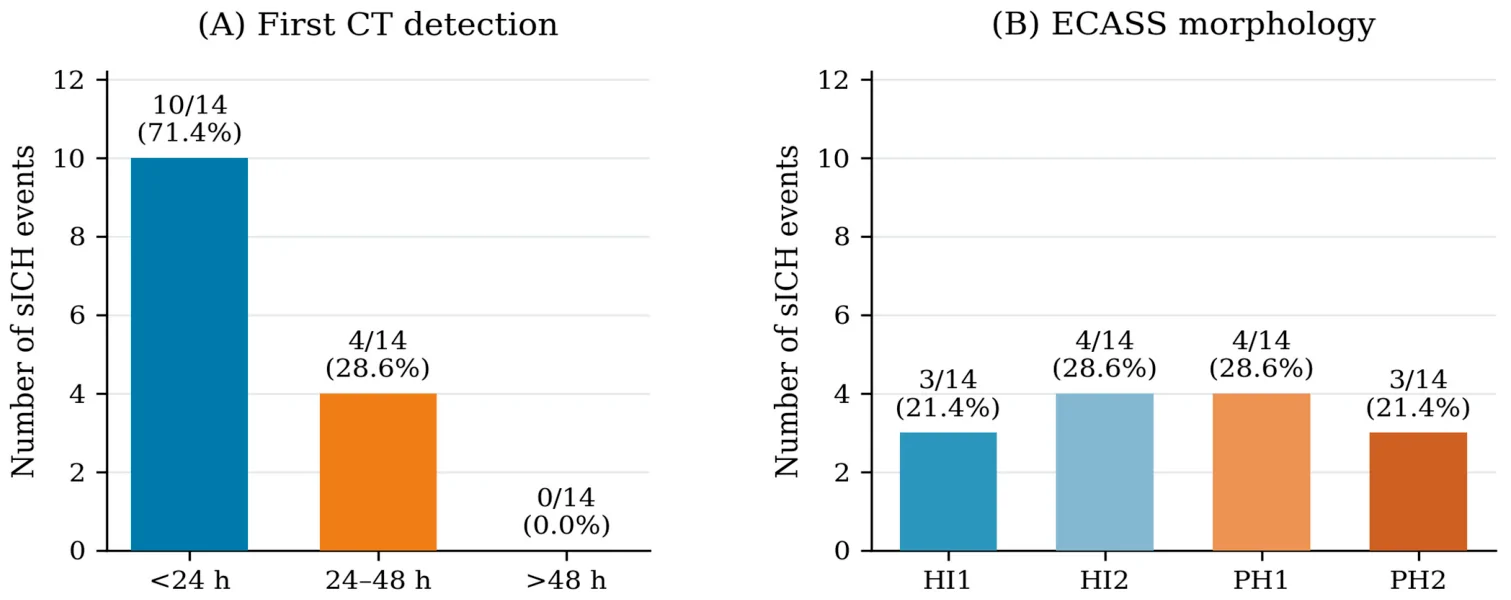
Detection timing (**A**) and ECASS morphology (**B**) among the 14 sICH events. Detection timing is presented in the validated aggregate windows of <24 h, 24–48 h, and >48 h after alteplase administration. The panels are separate distributions and do not link individual timing categories to morphology. ECASS, European Cooperative Acute Stroke Study; HI, hemorrhagic infarction; PH, parenchymal hematoma; sICH, symptomatic intracranial hemorrhage.

The validated aggregate detection windows and ECASS morphological distribution of the 14 study-defined sICH events are presented as separate panels in Figure 2.

Ten events (71.4%) were detected within 24 h and four (28.6%) in 24–48 h; no new hemorrhage was documented after 48 h. ECASS morphology comprised HI1 in three patients, HI2 in four, PH1 in four, and PH2 in three.

Ten sICH cases met the ≥4-point NIHSS deterioration criterion, whereas the remaining four had NIHSS increases of 1–3 points with documented hemorrhage-attributed deterioration involving consciousness, language, motor function, or cognition. No remote hemorrhage was identified.

### 3.3. Baseline Clinical and Neurological Characteristics

**Figure 3:**
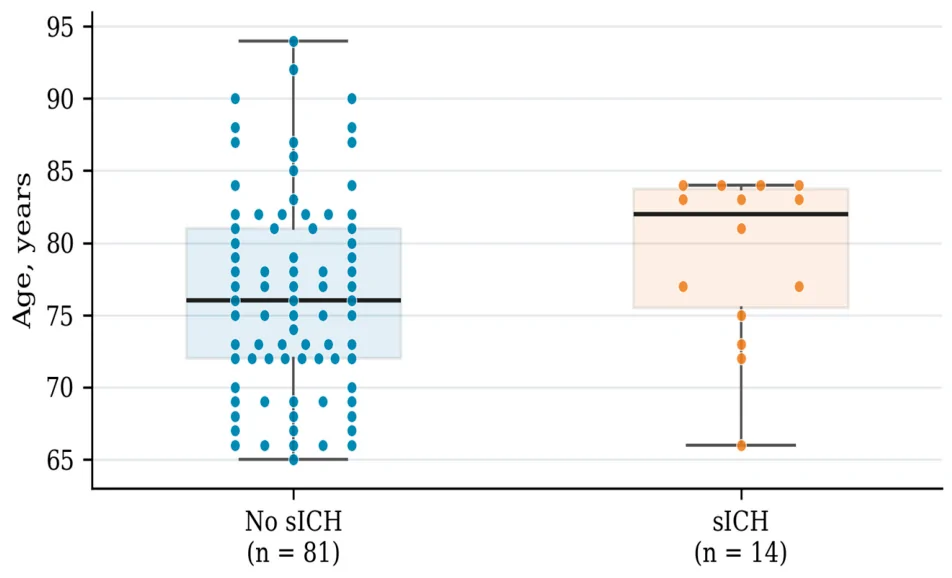
Individual ages according to sICH status. Each point represents one patient. Boxes show the median and interquartile range; whiskers extend to the most extreme values within 1.5 interquartile ranges. All ages are displayed, including values outside the whiskers. Horizontal spreading separates coincident ages. sICH, symptomatic intracranial hemorrhage. Blue denotes the no-sICH group, orange denotes the sICH group, and the black horizontal line within each box indicates the median.

Because individual ages provide information about dispersion and overlap that is not captured by summary statistics alone, the patient-level age distribution according to sICH status is shown in Figure 3.

The sICH group was descriptively older, although the distributions overlapped and the exploratory between-group comparison remained inconclusive. Exact medians, interquartile ranges, the median difference, and its 95% confidence interval are reported with the remaining baseline comparisons below.

Exact baseline demographic, neurological, vascular-territory, cardiovascular, treatment-exposure, and lifestyle comparisons, together with unadjusted effect estimates and 95% confidence intervals, are presented in Table 1.

Baseline comparisons were exploratory. Female sex showed the largest unadjusted association with sICH, while atrial fibrillation was also more frequent in the sICH group; however, the corresponding confidence intervals were wide.

Age and admission NIHSS were descriptively higher with sICH, but their confidence intervals included the null value. The remaining baseline comparisons were similarly imprecise and did not establish independent associations.

Within the sICH group, admission NIHSS was 15.0 [12.5–15.75] in women (*n* = 10) and 12.0 [10.25–15.0] in men (*n* = 4), with a median difference of 3.0 points (bootstrap 95% CI −6.0 to 7.0; exploratory Mann–Whitney *p* = 0.392).

### 3.4. In-Hospital Course

Median hospitalization was 14.0 [12.25–17.5] days with sICH and 9.0 [6.0–12.0] days without sICH. The descriptive median difference was 5.0 days (bootstrap 95% CI 3.0–9.0; *p* < 0.001).

In-hospital mortality occurred in 4/14 patients with sICH (28.6%) and 21/81 without sICH (25.9%). The absolute difference was 2.6 percentage points (conservative exact 95% CI −31.6 to 45.9), with an exact conditional OR of 1.14 (95% CI 0.24–4.52; Fisher *p* = 1.000).

### 3.5. Exploratory Multivariable Analyses

Exploratory adjusted associations were estimated in Model A using age, female sex, and atrial fibrillation, while Model B additionally incorporated admission NIHSS; the complete estimates are presented in Table 2.

All 95% profile-likelihood confidence intervals included the null value. Female sex produced the largest point estimate in both models, but neither estimate reached conventional statistical significance. Adding admission NIHSS did not materially change the female-sex point estimate, and neither model established an independent risk factor for sICH.

## 4. Discussion

### 4.1. Principal Findings

In this cohort of older alteplase-treated adults, the study-defined sICH frequency was 14.7%. All events were detected within 48 h, with representation of both hemorrhagic infarction and parenchymal hematoma. The endpoint combined radiographic confirmation with clinically attributed neurological deterioration.

Baseline group differences were descriptive. The female-sex imbalance remained hypothesis-generating after adjustment for age, AF, and admission NIHSS; neither model established an independent association. Hospitalization was longer with sICH, while the mortality difference was imprecise.

### 4.2. Frequency, Timing, and Morphological Pattern of sICH

The observed frequency is higher than many rates reported in broader alteplase-treated populations. Comparisons require the same hemorrhage definition, comparable patient selection, and similar surveillance [6]. The present endpoint required symptomatic deterioration, while ECASS terminology described morphology. Thus, the 14.7% estimate should be interpreted as the frequency of this study-defined clinical-imaging outcome in a selected older cohort, rather than as a directly standardized ECASS II or SITS-MOST rate.

Admission NIHSS characterized baseline neurological severity and was incorporated into Model B. The exploratory within-sICH comparison of admission NIHSS did not establish a sex difference in neurological severity. Infarct volume was not measured, and the present data therefore cannot establish whether infarct burden differed by sex or contributed to the observed hemorrhage frequency.

The early detection pattern is consistent with Chen et al., who reported a median time to symptomatic hemorrhage of approximately 18 h and identified the first 24 h as the period of greatest observed risk after alteplase [17]. The current distribution supports continued attention to neurological changes during early post-treatment monitoring, including the second day.

Symptomatic deterioration was not restricted to parenchymal hematoma. Clinical attribution and hemorrhagic morphology therefore provide complementary information [6]. In the four cases below the NIHSS threshold, the classification rested on documented clinically relevant deterioration attributed to bleeding. Petechial hemorrhage was not assigned hemorrhage-related mass effect.

### 4.3. Baseline Characteristics and Exploratory Associations

Age, admission NIHSS, AF, and early ischemic imaging abnormalities recur in evidence syntheses of post-thrombolysis hemorrhage [10]. Reviews of prediction models identify age and NIHSS as common predictors, while reporting substantial risk of bias [11]. Models A/B did not establish independent associations for their included covariates.

The unadjusted female-sex comparison had a nominal *p* value of 0.038, but it was one of multiple exploratory comparisons. Its wide interval and the inconclusive Firth estimates require interpretation as an imbalance for further investigation, rather than evidence of a sex-specific biological response to alteplase.

This interpretation is consistent with the absence of an overall sex difference in sICH in a thrombolysis meta-analysis [12]. Cohort and trial analyses also show that sex-associated outcome patterns accompany differences in age, neurological severity, comorbidity, and treatment characteristics [13,14]. Adjustment for admission NIHSS helps address neurological severity but does not resolve differences in infarct burden or other treatment-related factors.

AF was more frequent in the sICH group, but the adjusted intervals remained inconclusive. Observational and pooled reperfusion studies have associated AF with hemorrhagic outcomes [15,18]. The present estimate cannot distinguish the condition itself from accompanying cardioembolic features, infarct burden, or treatment characteristics.

### 4.4. Hospitalization and Mortality

Longer hospitalization with sICH may reflect additional monitoring, imaging, treatment changes, and complications. Length of stay describes the hospital course and does not isolate a causal consequence of hemorrhage. Hemorrhagic transformation has also been related to adverse clinical course in non-reperfusion cohorts [19], but that evidence cannot be treated as an alteplase-specific effect estimate.

The conservative exact mortality interval was compatible with clinically important differences in either direction. Four deaths among 14 sICH cases provide very limited power to detect a mortality difference and cannot establish similarity or equivalence between groups.

In an 836-patient multicenter cohort of endovascularly treated patients with disability at discharge, Mofatteh et al. identified age, baseline NIHSS, complete recanalization, and absence of parenchymal hematoma as independent predictors of three-month functional outcome [20]. Although its treatment setting and selected survivors differ from this alteplase cohort, the study supports the inclusion of neurological severity, reperfusion status, hemorrhage subtype, and follow-up functional outcomes in future analyses.

### 4.5. Clinical Interpretation, Strengths, and Limitations

These findings do not justify changing alteplase eligibility according to sex, age, AF, or any other isolated characteristic. Guideline-based clinical, imaging, and anticoagulant assessment remains necessary [1,2,5]. The aggregate DOAC findings do not establish safety after recent intake and cannot support a change in eligibility criteria.

The cohort was defined consecutively, deduplicated at the patient level, and restricted to alteplase administered at the study center. All patients underwent follow-up CT within 48 h. The 14 hemorrhage cases underwent clinical review and independent morphological assessment by two neurologists, and admission NIHSS was incorporated into descriptive and sensitivity analyses.

The retrospective single-center design, selected treated population, and 14-event count limit precision and generalizability. Firth penalization reduces small-sample estimation bias but does not eliminate residual confounding. Infarct volume was not measured. Large-vessel occlusion, collateral status, mechanical thrombectomy, stroke mechanism, baseline blood pressure, laboratory measures, actual doses and dose deviations, treatment intervals, pre-stroke disability, and discharge destinations were unavailable in a verifiable patient-linked form. These gaps prevented additional covariate adjustment, sensitivity analyses for glucose or thrombectomy, and a competing-event analysis of discharge. The direction and magnitude of residual confounding cannot be determined.

Anticoagulant detail was available only in aggregate form. Nine of 17 DOAC users had a reported last-dose interval ≤48 h, and drug-specific activity assays were unavailable. Standard INR or aPTT cannot establish the absence of clinically relevant DOAC activity [1]. The aggregate records did not allow independent verification of individual last-dose histories or eligibility decisions for these nine patients. Full concordance with guideline eligibility criteria therefore could not be established. Anticoagulant timing could not be linked to individual hemorrhage outcomes, so its contribution to the observed frequency could not be estimated.

The four sICH classifications below the ≥4-point NIHSS threshold depended on retrospective documentation of clinical attribution. Detailed adjudication focused on recorded hemorrhage-positive cases, leaving possible ascertainment error elsewhere. Imaging was routinely scheduled but actual examination times varied by several hours; detection windows therefore do not establish the exact time of hemorrhage onset. AF combined pre-existing and newly detected diagnoses, and chart-based lifestyle classifications may be inconsistent. Hospitalization is also affected by death, discharge destination, rehabilitation access, and local practice.

The study-defined sICH endpoint is not interchangeable with NINDS, ECASS II, or SITS-MOST definitions [6]. Post-discharge functional outcomes were not analyzed, and mortality estimates were underpowered. These findings warrant prospective multicenter assessment using harmonized outcome definitions, detailed baseline imaging, verified anticoagulant timing, reperfusion characteristics, and longer-term functional follow-up.

## 5. Conclusions

Among 95 older adults treated with intravenous alteplase, 14 recorded hemorrhages met the study-defined sICH criteria. Detection occurred within 48 h, predominantly before 24 h, and included hemorrhagic infarction and parenchymal hematoma subtypes.

Adjusted association estimates were inconclusive, including after adjustment for admission NIHSS. Infarct volume was not measured. sICH was descriptively associated with longer hospitalization, while the mortality difference remained imprecise. The findings do not establish independent risk factors or justify changes to guideline-based eligibility.

Larger prospective multicenter studies should use standardized hemorrhage definitions, detailed stroke-severity and imaging measures, verified treatment information, and follow-up functional outcomes.

## Figures and Tables

**Table 1:** Baseline clinical and neurological characteristics according to sICH status.

Characteristic	Overall *n* = 95	No sICH *n* = 81	sICH *n* = 14	Unadjusted Effect Estimate (95% CI)	*p* Value
Age, years	77.0 [72.0–82.0]	76.0 [72.0–81.0]	82.0 [75.5–83.8]	Median difference 6.0 (−1.0 to 9.0)	0.110
Admission NIHSS	12.0 [10.0–16.0]	12.0 [10.0–16.0]	14.5 [12.0–15.75]	Median difference 2.5 (−1.0 to 4.0)	0.166
Previous stroke	12 (12.6%)	11 (13.6%)	1 (7.1%)	OR 0.49 (0.01–3.97)	0.687
Carotid territory, recorded	91 (95.8%)	77 (95.1%)	14 (100.0%)	OR ∞ (0.11–∞)	1.000
Vertebrobasilar territory, recorded	3 (3.2%)	3 (3.7%)	0 (0.0%)	OR 0.00 (0.00–14.52)	1.000
Female sex	41 (43.2%)	31 (38.3%)	10 (71.4%)	OR 3.97 (1.03–18.89)	0.038
Urban residence	63 (66.3%)	54 (66.7%)	9 (64.3%)	OR 0.90 (0.24–3.77)	1.000
Acute myocardial infarction	9 (9.5%)	8 (9.9%)	1 (7.1%)	OR 0.70 (0.01–6.07)	1.000
Congestive heart failure	31 (32.6%)	25 (30.9%)	6 (42.9%)	OR 1.67 (0.43–6.17)	0.374
Transient ischemic attack	3 (3.2%)	3 (3.7%)	0 (0.0%)	OR 0.00 (0.00–14.52)	1.000
Diabetes mellitus	29 (30.5%)	23 (28.4%)	6 (42.9%)	OR 1.88 (0.48–6.98)	0.348
Arterial hypertension	80 (84.2%)	67 (82.7%)	13 (92.9%)	OR 2.69 (0.35–123.57)	0.457
Atrial fibrillation	35 (36.8%)	27 (33.3%)	8 (57.1%)	OR 2.64 (0.72–10.24)	0.132
History of cancer	7 (7.4%)	7 (8.6%)	0 (0.0%)	OR 0.00 (0.00–4.14)	0.589
Prior anticoagulant therapy	24 (25.3%)	21 (25.9%)	3 (21.4%)	OR 0.78 (0.13–3.36)	1.000
Prior antiplatelet therapy	42 (44.2%)	34 (42.0%)	8 (57.1%)	OR 1.83 (0.50–7.04)	0.385
Both prior antithrombotic classes	22 (23.2%)	19 (23.5%)	3 (21.4%)	OR 0.89 (0.14–3.87)	1.000
Any prior antithrombotic therapy	44 (46.3%)	36 (44.4%)	8 (57.1%)	OR 1.66 (0.46–6.37)	0.401
Smoking	7 (7.4%)	6 (7.4%)	1 (7.1%)	OR 0.96 (0.02–9.02)	1.000
Alcohol use	15 (15.8%)	13 (16.0%)	2 (14.3%)	OR 0.87 (0.09–4.69)	1.000
Obesity	19 (20.0%)	16 (19.8%)	3 (21.4%)	OR 1.11 (0.18–4.91)	1.000

Data are median [IQR] or *n* (%). Median differences (sICH minus no sICH) have percentile bootstrap 95% CIs (10,000 resamples); ORs and CIs are exact conditional estimates. *p* values use Mann–Whitney U or Fisher exact tests; comparisons are exploratory. Both-class exposure denotes positive flags for both antithrombotic classes. NIHSS, National Institutes of Health Stroke Scale; IQR, interquartile range; OR, odds ratio; CI, confidence interval; sICH, symptomatic intracranial hemorrhage; ∞, unbounded.

**Table 2:** Exploratory Firth penalized logistic regression models for sICH.

Predictor	OR	95% Profile-Likelihood CI	Profile LR *p* Value
Model A. Exploratory model			
Female sex	3.001	0.918–11.195	0.069
Age, per 10-year increase	1.202	0.479–3.020	0.691
Atrial fibrillation	1.776	0.516–6.274	0.360
Model B. Additional adjustment for admission NIHSS			
Female sex	3.069	0.943–11.418	0.063
Age, per 10-year increase	1.162	0.473–2.852	0.740
Atrial fibrillation	1.565	0.449–5.563	0.479
Admission NIHSS, per 5-point increase	1.326	0.711–2.472	0.367

Both models included 95 patients and 14 sICH events. Firth logistic regression used Jeffreys-prior penalization. CIs are profile penalized likelihood intervals; *p* values derive from the corresponding likelihood-ratio test. Age and admission NIHSS were continuous covariates. CI, confidence interval; LR, likelihood ratio; NIHSS, National Institutes of Health Stroke Scale; OR, odds ratio; sICH, symptomatic intracranial hemorrhage.

## Data Availability

The patient-level data supporting the findings of this study are not publicly available because they were derived from hospital medical records and are subject to patient confidentiality, ethical restrictions, and institutional data-protection requirements. Requests for de-identified data should be directed to the corresponding authors and will be considered subject to the required institutional and ethical approvals.

## Supplementary Materials

The following supporting information can be downloaded at: https://www.mdpi.com/article/10.3390/neurolint18100194/s1, STROBE Statement Checklist.

## Abbreviations

The following abbreviations are used in this manuscript:

AF	Atrial fibrillation
AIS	Acute ischemic stroke
aPTT	Activated partial thromboplastin time
BMI	Body mass index
CI	Confidence interval
CT	Computed tomography
DOAC	Direct oral anticoagulant
ECASS	European Cooperative Acute Stroke Study
eGFR	Estimated glomerular filtration rate
GCS	Glasgow Coma Scale
HI	Hemorrhagic infarction
INR	International normalized ratio
IQR	Interquartile range
IV	Intravenous
NIHSS	National Institutes of Health Stroke Scale
NINDS	National Institute of Neurological Disorders and Stroke
OR	Odds ratio
PH	Parenchymal hematoma
sICH	Symptomatic intracranial hemorrhage
SITS-MOST	Safe Implementation of Thrombolysis in Stroke-Monitoring Study
STROBE	Strengthening the Reporting of Observational Studies in Epidemiology
