# Supplementary material for: Clinical Characteristics and In-Hospital Course of Symptomatic Intracranial Hemorrhage After Intravenous Alteplase in Older Adults with Acute Ischemic Stroke: A Retrospective Cohort Study
Source: Neurol Int. 2026 Oct 15;18(10):194. doi: 10.3390/neurolint18100194 (PMC13708256; doi:10.3390/neurolint18100194)
Supplement: Supplementary file 1 [file neurolint-18-00194-s001.zip › neurolint-4578178-supplementary.pdf]

STROBE Statement—Checklist

Clinical Characteristics and In-Hospital Course of Symptomatic Intracranial Hemorrhage After Intravenous Alteplase in Older Adults With Acute Ischemic Stroke: A Retrospective Cohort Study

	Item No	Recommendation	Reported on page #
**Title and abstract**	1	(*a*) Indicate the study's design with a commonly used term in the title or the abstract	p. 1, title and Abstract: retrospective single-center cohort.
		(*b*) Provide in the abstract an informative and balanced summary of what was done and what was found	p. 1, structured Abstract: setting, methods, study-defined sICH, main estimates, uncertainty, and conclusions.
**Introduction**			
Background/rationale	2	Explain the scientific background and rationale for the investigation being reported	pp. 1–2, Introduction: clinical context and evidence gap.
Objectives	3	State specific objectives, including any prespecified hypotheses	p. 2, final Introduction paragraph; pp. 2–3, Section 2.2; p. 5, Section 2.7. The primary descriptive analysis has no directional hypothesis. Exploratory secondary objectives are identified, and Model A/B covariates are described as selected before examination of the study results.
**Methods**			
Study design	4	Present key elements of study design early in the paper	p. 2, Section 2.1: retrospective design and index hospitalization.
Setting	5	Describe the setting, locations, and relevant dates, including periods of recruitment, exposure, follow-up, and data collection	p. 2, Section 2.1; p. 3, Section 2.3; p. 6, Section 2.8. Center, 2020–2024 care period, monitoring, and post-approval research extraction in July 2026.
Participants	6	(*a*) Give the eligibility criteria, and the sources and methods of selection of participants. Describe methods of follow-up	pp. 2–3, Section 2.2: consecutive source admissions, age/treatment eligibility, transfers, deduplication, and index

			selection. p. 6, Figure 1; p. 3, Section 2.3: follow-up imaging.
		(*b*) For matched studies, give matching criteria and number of exposed and unexposed	Not applicable: this was not a matched cohort.
Variables	7	Clearly define all outcomes, exposures, predictors, potential confounders, and effect modifiers. Give diagnostic criteria, if applicable	pp. 3–4, Sections 2.4–2.5; p. 5, Section 2.7. Outcome, clinical definitions, exposures, baseline NIHSS, and model covariates are described.
Data sources/ measurement	8*	For each variable of interest, give sources of data and details of methods of assessment (measurement). Describe comparability of assessment methods if there is more than one group	pp. 3–5, Sections 2.3–2.6: routine-care sources, imaging review, clinical definitions, coding, restriction to patient-linked variables, and descriptive use of aggregate anticoagulant summaries.
Bias	9	Describe any efforts to address potential sources of bias	pp. 2–5, Sections 2.2 and 2.5–2.7; pp. 11–12, Section 4.5. Deduplication, independent CT review, restriction to variables with verifiable patient linkage, parsimonious Firth models, and remaining bias are described.
Study size	10	Explain how the study size was arrived at	pp. 2–3, Section 2.2: all eligible patients analyzed; no prospective sample-size calculation.
Quantitative variables	11	Explain how quantitative variables were handled in the analyses. If applicable, describe which groupings were chosen and why	pp. 3–4, Section 2.4; p. 5, Section 2.7. Age and admission NIHSS are analyzed continuously with stated scaling; other recorded clinical variables are handled according to their documented coding.
Statistical methods	12	(*a*) Describe all statistical methods, including those used to control for confounding	p. 5, Section 2.7: Mann–Whitney/Fisher tests, exact conditional ORs, bootstrap median differences, Wilson frequency interval, conservative exact mortality

			difference interval, and Firth models.
		(*b*) Describe any methods used to examine subgroups and interactions	p. 5, Section 2.7; p. 9, Section 3.3: exploratory admission-NIHSS comparison between women and men within sICH. No formal interaction analysis was performed.
		(*c*) Explain how missing data were addressed	p. 5, Section 2.6: reported patient-level variables used in Table 1, hospital-course comparisons, and Models A/B were complete in 95 patients; no imputation was used. Variables from a separate clinical extract without verifiable Study_ID linkage were excluded. p. 7, Section 3.1: available anticoagulant last-dose timing counts are reported only in aggregate form.
		(*d*) If applicable, explain how loss to follow-up was addressed	p. 2, Section 2.1; p. 9, Section 3.4. Follow-up was restricted to the index hospitalization; no post-discharge follow-up analysis was performed.
		(*e*) Describe any sensitivity analyses	p. 5, Section 2.7; pp. 9–10, Section 3.5 and Table 2: Model B adds admission NIHSS. Other unavailable sensitivity analyses are identified in Section 4.5, pp. 11–12.
**Results**			
Participants	13*	(a) Report numbers of individuals at each stage of study—eg numbers potentially eligible, examined for eligibility, confirmed eligible, included in the study, completing follow-up, and analysed	pp. 2–3, Section 2.2; pp. 6–7, Section 3.1; p. 6, Figure 1: 200 hospitalizations to 95 unique analyzed patients.
		(b) Give reasons for non-participation at each stage	pp. 2–3, Section 2.2; p. 6, Figure 1: age <65 years, no alteplase, and repeat-

			record removals.
		(c) Consider use of a flow diagram	p. 6, Figure 1: cohort flow diagram.
Descriptive data	14*	(a) Give characteristics of study participants (eg demographic, clinical, social) and information on exposures and potential confounders	pp. 6–9, Sections 3.1 and 3.3; pp. 8–9, Table 1. NIHSS, recorded territory, clinical history, and antithrombotic exposure summaries are reported; unavailable or unlinked variables are disclosed in Section 4.5.
		(b) Indicate number of participants with missing data for each variable of interest	p. 5, Section 2.6: core patient-level variables used in the reported analyses were complete in 95 patients. p. 7, Section 3.1: exact anticoagulant last-dose timing was available in 18/24 anticoagulant-exposed patients and in 11/17 DOAC users; these details were aggregate only.
		(c) Summarise follow-up time (eg, average and total amount)	p. 9, Section 3.4: group-specific hospitalization medians and IQRs. Observation was the index hospital stay; no longitudinal post-discharge follow-up.
Outcome data	15*	Report numbers of outcome events or summary measures over time	pp. 6–7, Sections 3.1–3.2 and Figure 2: 14 study-defined sICH events, symptom criteria, detection windows, and ECASS morphology. p. 9, Section 3.4: hospitalization and deaths.
Main results	16	(*a*) Give unadjusted estimates and, if applicable, confounder-adjusted estimates and their precision (eg, 95% confidence interval). Make clear which confounders were adjusted for and why they were included	pp. 8–9, Table 1; p. 9, Section 3.4; pp. 9–10, Table 2. Unadjusted effects, adjusted ORs, 95% CIs, and exact mortality difference. Covariate rationale: Section 2.7, p. 5.
		(*b*) Report category boundaries when continuous variables were categorized	pp. 2–4, Sections 2.2 and 2.4: ≥65-year

			inclusion threshold, continuous age per 10 years, and admission NIHSS per 5 points; no model categorization.
		(*c*) If relevant, consider translating estimates of relative risk into absolute risk for a meaningful time period	pp. 6–7, Section 3.1: absolute sICH frequency. p. 9, Section 3.4: absolute in-hospital mortality proportions and risk difference with conservative exact 95% CI.
Other analyses	17	Report other analyses done—eg analyses of subgroups and interactions, and sensitivity analyses	p. 9, Section 3.3: exploratory within-sICH NIHSS comparison by sex. pp. 9–10, Section 3.5 and Table 2: Model B adds admission NIHSS. No formal interactions or prediction validation were performed.
**Discussion**			
Key results	18	Summarise key results with reference to study objectives	p. 10, Section 4.1; p. 12, Conclusions.
Limitations	19	Discuss limitations of the study, taking into account sources of potential bias or imprecision. Discuss both direction and magnitude of any potential bias	pp. 11–12, Section 4.5: selection, attribution and detection uncertainty, 14-event precision, unlinked variables, unavailable sensitivity analyses, DOAC eligibility uncertainty, and hospital-course limitations. The direction and magnitude of residual confounding cannot be determined.
Interpretation	20	Give a cautious overall interpretation of results considering objectives, limitations, multiplicity of analyses, results from similar studies, and other relevant evidence	pp. 10–12, Discussion: endpoint definition, exploratory estimates, multiplicity, earlier evidence, clinical interpretation, and limitations.
Generalisability	21	Discuss the generalisability (external validity) of the study results	pp. 11–12, Section 4.5; p. 12, Conclusions: single-center treated older population and need for multicenter

			confirmation.
**Other information**			
Funding	22	Give the source of funding and the role of the funders for the present study and, if applicable, for the original study on which the present article is based	p. 13, Funding statement: no external funding; doctoral research context.

*Give information separately for exposed and unexposed groups.

Note: An Explanation and Elaboration article discusses each checklist item and gives methodological background and published examples of transparent reporting. The STROBE checklist is best used in conjunction with this article (freely available on the Web sites of PLoS Medicine at http://www.plosmedicine.org/, Annals of Internal Medicine at http://www.annals.org/, and Epidemiology at http://www.epidem.com/). Information on the STROBE Initiative is available at http://www.strobe-statement.org.
